# Progress and bottleneck in induced pluripotency

**DOI:** 10.1186/2045-9769-1-5

**Published:** 2012-07-06

**Authors:** Zhen-Ning Zhang, Yang Xu

**Affiliations:** Section of Molecular Biology, Division of Biological Sciences, University of California, San Diego, 9500 Gilman Drive, La Jolla California 92093-0322 USA

## Abstract

With their capability to undergo unlimited self-renewal and to differentiate into all cell types in the body, induced pluripotent stem cells (iPSCs), reprogrammed from somatic cells of individual patients with defined factors, have unlimited potential in cell therapy and in modeling complex human diseases. Significant progress has been achieved to improve the safety of iPSCs and the reprogramming efficiency. To avoid the cancer risk and spontaneous reactivation of the reprogramming factors associated with the random integration of viral vectors into the genome, several approaches have been established to deliver the reprogramming factors into the somatic cells without inducing genetic modification. In addition, a panel of small molecule compounds, many of which targeting the epigenetic machinery, have been identified to increase the reprogramming efficiency. Despite these progresses, recent studies have identified genetic and epigenetic abnormalities of iPSCs as well as the immunogenicity of some cells derived from iPSCs. In addition, due to the oncogenic potential of the reprogramming factors and the reprogramming-induced DNA damage, the critical tumor suppressor pathways such as p53 and ARF are activated to act as the checkpoints that suppress induced pluripotency. The inactivation of these tumor suppression pathways even transiently during reprogramming processes could have significant adverse impact on the genome integrity. These safety concerns must be resolved to improve the feasibility of the clinic development of iPSCs into human cell therapy.

## Somatic nuclear reprogramming

The pluripotent cells in the inner cell mass (ICM) are able to differentiate into each cell type of the three germ layers [1], and give rise to embryonic stem (ES) cell lines [2, 3]. Considering their unlimited self-renewal capability and pluripotency to differentiate into all cell types in the body, human pluripotent stem cells (hESCs) hold great promise in human cell therapy. However, one major drawback of the hESC-based cell therapy is the allogenic immune rejection of hESC-derived cells by the recipients. While persistent systemic immune suppression could prevent the rejection of the allogenic hESC-derived cells, the long-term immunosuppression has serious side effects such as the increased risk for cancer and infection [4]. Therefore, it would be highly desirable to develop pluripotent cells with the same genetic makeup as the patients, as the cells derived from the patient-specific pluripotent cells are considered autologous and thus can be transplanted without the risk of immune rejection.

While it had been assumed that differentiating cells gradually lose their plasticity during development and the terminally differentiated cells could not give rise to another cell type, this assumption has been challenged by the discovery of nuclear reprogramming that allows the switch of cell identity from one cell type to another. Somatic nuclear reprogramming to convert a somatic nucleus into the pluripotent state was first described in 1950s when researchers transferred nuclei from amphibian blastula into enucleated eggs, resulting in adult individuals [5, 6]. In subsequent experiments, this somatic cell nuclear transfer (SCNT) technology could achieve the same results with the nucleus from more differentiated cells such as intestinal cells and skin cells [7, 8]. One of the most exciting developments in the somatic nuclear reprogramming is the first success in mammalian SCNT in 1997 to produce the cloned sheep Dolly by transferring the nuclei of adult mammary gland cells into enucleated eggs [9]. Since then, SCNT has been successfully applied to many other mammalian species, including mice and nonhuman primate [10–16]. Successful SCNT in human has recently been reported [17]. In this context, the nucleus from human somatic cells can be reprogrammed into pluripotent state after transferring into human eggs, but the nucleus of the human egg must be retained for the derivation of pluripotent stem cells from the cloned embryos. In summary, these results demonstrate that the somatic genome has the potential to be reprogrammed into pluripotent state.

The efficiency of SCNT to generate cloned embryos declines dramatically when the nucleus is derived from more differentiated cells [18]. In addition, the cloned animals often exhibit phenotypic and genetic abnormalities [15, 19–21]. Another approach for somatic nuclear reprogramming is through cell fusion when the nucleus of the one fusion partner can be reprogrammed into the epigenetic state of the other fusion partner [22]. In this context, when fused with pluripotent stem cells, the nucleus of the somatic cells can be reprogrammed into the pluripotent state. For example, the fusion of thymocytes with embryonic carcinoma cells (ECCs) could generate immortalized pluripotent cell lines [23, 24], and the resulting hybrid cells acquired the properties of ECCs [25, 26]. Similarly, the fusion of mouse ES cells with thymocytes can lead to pluripotent hybrid cells [27–29]. In addition, the fusion of the human somatic cells with hESCs generates pluripotent hybrid cells, and the overexpression of Nanog can enhance such reprogramming efficiency [30, 31]. While these studies further confirm the feasibility to reprogram somatic nucleus into pluripotent state, the presence of two sets of genome in the hybrid cells remains an obstacle for any application of these pluripotent stem cells.

Due to the technical difficulties and the ethic concerns with the usage of human eggs, it remains a major challenge to generate human patient’s specific pluripotent stem cells. The groundbreaking discovery of the induced pluripotency with defined factors by Yamanaka and colleagues has revolutionized the field of somatic nuclear reprogramming. By screening two dozens factors that are expressed in ESCs, they discovered that the combination of four transcription factors (Oct4, Sox2, Klf4 and c-Myc) could reprogram mouse fibroblasts into pluripotent stem cells, termed induced pluripotent stem cells (iPSCs) [32]. Like ESCs, iPSCs are capable of unlimited self-renewal and can differentiate into each cell type of the three germ layers. Soon afterwards, the iPSC technology was used successfully to reprogram somatic cells from a rapidly growing list of species into iPSCs, including human [33–35], monkey [36], rat [37]. In addition, somatic cells can also be reprogrammed into iPSCs with different combinations of reprogramming factors [34, 38]. Even the terminally differentiated cells can be successfully reprogrammed into iPSCs, although the efficiency is much lower than the reprogramming of precursor cell types [39–41]. The discovery of iPSC technology greatly improves the feasibility in developing patient-specific cell therapy and provides the unique opportunity in modeling human diseases.

## Progress in iPSC biology

The initial reprogramming factors discovered by Yamanaka and colleagues are Oct4, Sox2, Klf4 and c-Myc, which can reprogram somatic cells of various species into iPSCs [32, 33]. Another set of reprogramming factors (Oct4, Sox2, Nanog, and Lin28) can also reprogram mouse and human somatic cells into iPSCs [34, 42]. c-Myc is a potent oncogene [43]. Therefore, to reduce the oncogenic potential of iPSCs, c-Myc can be excluded from the reprogramming cocktail but with much lower efficiency [37, 44–49]. The requirement for various reprogramming factors depends on the progenitor cell types. For example, Oct4 and Sox2 alone are sufficient to reprogram the cord blood cells into iPSCs [50]. Oct4 and Klf4 alone are sufficient to reprogram adult mouse neural stem cells and dermal papilla cells into iPSCs [51, 52]. Oct4 alone is sufficient to reprogram the neural stem cells into iPSCs possibly due to the high level of endogenous expression of the other reprogramming factor such as Sox2 [53, 54].

Beside Oct4, Sox2, Klf4 and c-Myc or Nanog, and Lin28, there are other modulators that can either substitute for or work together with them to improve reprogramming efficiency (Table 1). Indeed, these modulators are divided into several categories. One group falls into transcription factors. Orphan nuclear receptor such as Esrrb [55] and Nr5a2 [56] could replace Klf4 and Oct4 respectively and mediate reprogramming of MEFs. Other transcription factors like Sall4 [57], CCAAT/enhancer-binding-protein-α (C/EBPα) [58], UTF-1, an ESC-specific transcription factor increases reprogramming efficiency [59] are also reported to improve reprogramming. Another class comprises cell signaling and proliferation modulators. Overexpress SV40 large T antigen (SV40 LT) [60] or human telomerase (hTERT) [61], two proteins that promote proliferation in MEFs greatly increased the reprogramming efficiency. Some microRNAs function as cell cycle regulators also influence reprogramming [62, 63]. It has been reported that TGFβ, bone morphogenetic proteins (BMPs) and Wnt signaling pathways could also modulate reprogramming [64–68]. Epigenetic regulators are the other subfamily. Histone deacetylase [69–71], methyltransferase [72] and DNA methyltransferase [73, 74] have been implicated to influence reprogramming. As the list keeps growing, our understanding of the mechanism of reprogramming will go deeper and further. Novel modulators may provide new important targets for small molecules that would further increase the reprogramming efficiency in a safer manner.

**Table 1:** **Reprogramming modulators**

Category	Function	Reference
Transcription factor		
Esrrb(Orphan nuclear receptor )	replace Klf4	[55]
Nr5a2(Orphan nuclear receptor )	replace Oct4	[56]
Sall4	increase the efficiency of reprogramming	[57]
C/EBPα	reprogramming of mature B cells	[58]
UTF-1(ESC-specific transcription factor )	increase the efficiency of reprogramming	[59]
SV40 LT	increase the efficiency of reprogramming	[60]
miR-291-39, miR-294, miR-295	increase the efficiency of reprogramming	[62, 63]
miR-372, miR-302/367		[75, 76]
hTERT	increase the efficiency of reprogramming	[61]
TGFβ	antagonist increase the efficiency of reprogramming	[65–67]
Wnt3a	increase the efficiency of reprogramming	[68]
BMP4	replace Klf4	[64]
Epigenetic regulators		
HDACs	inhibition increase the efficiency of reprogramming	[69–71]
G9a	increase the efficiency of reprogramming	[72]
DNMT1	inhibition promote fully reprograming	[73, 74]

To optimize the transduction efficiency, the reprograming factors were initially delivered into cells using retroviral or lentiviral vectors that can be integrated randomly into the genome [77]. Because the random integration of the viral vectors in the genome of iPSCs pose a serious risk, significant effort was devoted to generate iPSCs without any genetic modification. In this context, adenoviral vector [78, 79], plasmid vector [80] and minicircle [81], episomal vectors [42, 82, 83], piggyBac transposon systems [84, 85], membrane-permeable reprogramming factors [86, 87], synthetic mRNA [88], MicroRNAs [63, 75, 76], have been used to reprogram somatic cells into integration-free iPSCs. However, the efficiency of these reprogramming technologies remains lower than the retroviral vector-based reprogramming approach [77]. This problem could be partially mitigated by the identification of small molecule chemical compounds that can significantly improve the reprogramming efficiency (Table 2). These compounds include the Valproic acid, trichostatin A (TSA) and sodium butyrate, all histone deacetylase inhibitors [70], BIX-01294, an inhibitor of histone methyl transferase (HMT) [89], Parnate, a histone demethylase inhibitor [90], 5-azacytidine (5-aza) and RG108, DNA methyltransferase inhibitors [74, 89]. All these chemicals are epigenetic modifiers, indicating the importance of epigenetic change during the reprogramming. Other small molecule compounds, such as the antagonist of the transforming growth factor β (TGFβ) pathway [67, 91], the activator of the 3-phosphoinositide-dependent protein kinase 1 (PDK1) [92] and Vitamin C [93] can also dramatically increase reprogramming efficiency. Therefore, the combination of these chemical biology and integration-free reprogramming strategies could significantly improve the efficiency to generate integration-free iPSCs.

**Table 2:** **Small molecules that promote reprogramming**

Compound	Function	Reference
Valproic acid	histone deacetylase inhibitor	[70]
Trichostatin A	histone deacetylase inhibitor	[70]
Sodium butyrate	histone deacetylase inhibitor	[70]
BIX-01294	histone methyl transferase inhibitor	[89]
Parnate	histone demethylase inhibitor	[90]
5-azacytidine	DNA methyltransferase inhibitor	[74]
RG108	DNA methyltransferase inhibitor	[89]
SB431542 + PD0325901	ALK5 inhibitor + MEK inhibitor	[67]
A-83-01	TGFβ receptor inhibitor	[92]
CHIR99021	GSK3 inhibitor	[67]
RepSox	Tgfbr1 kinase inhibitor	[65]
PS48	activator of PDK1	[92]
Vitamin C	nutrient vital that lower reactive oxygen species	[93]

## Bottlenecks of IPSCs

### Genetic and epigenetic instability and immunogenicity of iPSCs

**Figure 1:**
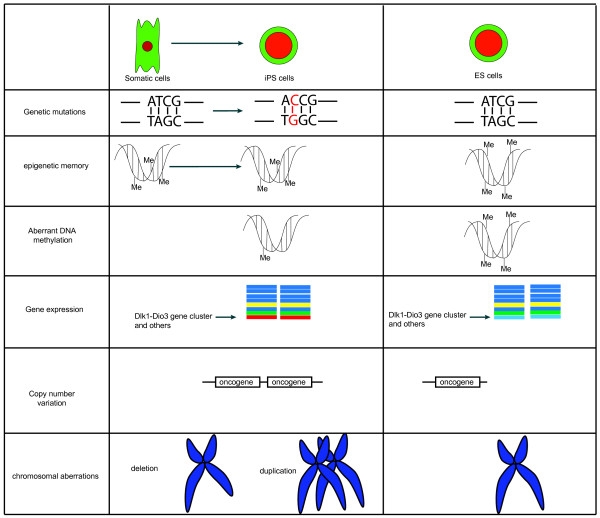
**Genetic and epigenetic abnormalities in iPSCs.** Induced pluripotency leads to genetic and epigenetic defects in iPSCs including genomic DNA mutation, abnormal genomic DNA methylation and gene expression, copy number variation and chromosomal aneuploidy.

When considering the clinic application of iPSC and ESCs, iPSCs appear to have several advantages over ESCs. For example, the generation of iPSCs avoids using human embryos, a major ethic concern for the generation of hESCs. The cells derived from patient-specific iPSCs are considered autologous cells and thus will not be rejected by the patient’s immune system. In addition, iPSCs derived from human patients offer the first opportunity to model human diseases with complex traits. Recent studies, however, have raised the concern of the safety of iPSCs in clinic application. While the global gene expression profile of iPSCs is very close to ESCs, there remains transcriptional signature that can distinguish between iPSCs and ESCs (Figure 1) [94]. Recent studies have also identified significant epigenetic differences between iPSCs and ESCs. By comparing genetically identical ESCs and iPSCs, it has been shown that expression levels for two genes (Gtl2 and Rian) and 21 miRNAs, all present on the imprinted Dlk1-Dio3 gene cluster on Chromosome 12qF1, differ significantly. Because of the developmental role of the Dlk1-Dio3 gene cluster, these iPSCs contributed poorly to chimaeras and failed to develop into adult animals with tetraploid complementation [95, 96]. In addition, iPSCs appear to retain some DNA methylation signatures of their somatic cells of origin, called epigenetic memory [97, 98]. The epigenetic memory could potentiate the gene expression during the iPSCs differentiation that favors the differentiation to the original lineage, while restricting the differentiation potential to other lineages [98]. In addition, by whole-genome profile of DNA methylation at the single-base resolution, recent studies have shown that iPSCs harbor both epigenetic memory and aberrant DNA methylation [99].

Recent studies have shown that iPSCs also harbor genetic mutations that are introduced during reprogramming [100, 101]. In addition, increased genetic abnormalities such as copy number variation (CNV) [102, 103], chromosomal aberrations [104] are detected in iPSCs, especially in the early passages of iPSCs. While it remains unclear how these genetic abnormalities impact on the reprogramming efficiency, some of the gene mutations are associated with human cancers [100]. Therefore, the cells derived from iPSCs could have increased cancer risk. In support of this notion, chimeric mice and tetraploid complemented mice generated with iPSCs reprogrammed with Oct4/Sox2/Klf4/c-Myc in viral vectors are highly susceptible to tumorigenesis [40, 105]. The cancer risk associated with integration-free iPSCs remains to be examined, especially when the highly oncogenic c-Myc and Klf4 are left out of the reprogramming cocktail.

**Figure 2:**
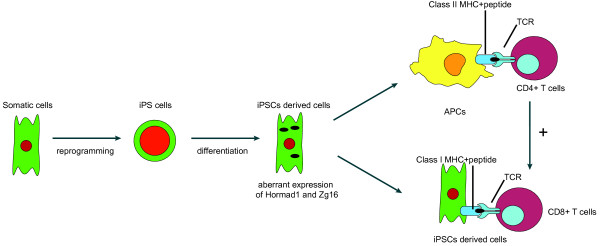
**The Immunogenicity of iPSC derivatives.** The abnormal overexpression of immunogenic proteins such as Hormad1 and Zg16 in iPSC-derived cells leads to the antigen-specific T cell activation. APC, antigen presenting cells; MHC, major histocompatibility complex; TCR, T cell receptor.

While it has been generally assumed that autologous cells derived from patient-specific iPSCs should be immune tolerated by the patient, it is possible that the genetic and epigenetic abnormalities of iPSCs could contribute to minor antigens in some hESC-derived cells. Several reports have shown that transplantation of iPSC-derived cells could ameliorate disease phenotypes in mouse models without apparently immune rejection [106, 107]. However, these studies were carried out in either immune privileged site or in lethally irradiated mice. Taking advantage of the capability of iPSCs to form teratomas that contain all lineages of cells in the body, recent studies have demonstrated that, unlike ESC-derived cells that are not immunogenic in syngeneic hosts, some cells derived from iPSCs are immunogenic in the syngeneic recipients due to the abnormal expression of minor antigens in some cells in the teratomas during the differentiation of iPSCs (Figure 2) [42]. While remaining to be confirmed, the abnormal overexpression of the minor antigens might be due to the abnormal epigenetics of iPSCs. In addition, the contribution of the coding sequence mutations to the immunogenicity of iPSC-derived cells remains to be examined.

### Checkpoints in induced pluripotency

**Figure 3:**
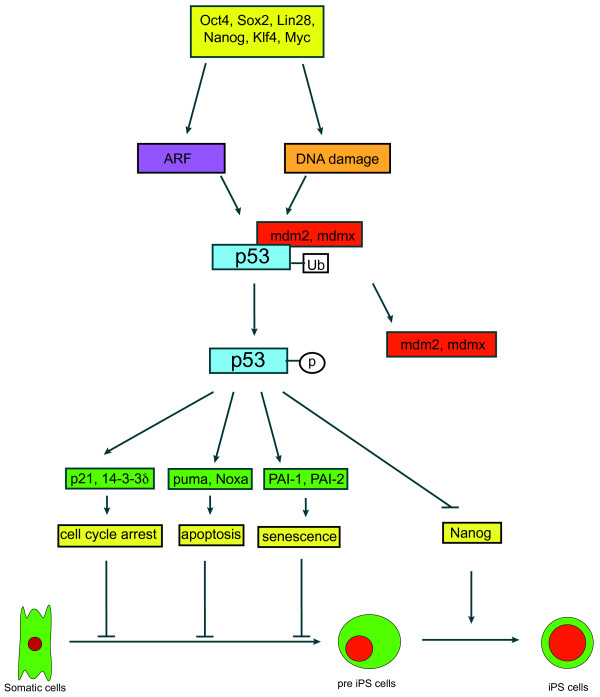
**Tumor suppressor p53 inhibits induced pluripotency.** p53 is activated by oncogenic stresses and DNA damage during reprogramming, leading to cell cycle arrest, apoptosis, or senescence, which all limit successful reprogramming. Upon activation, p53 also suppresses the expression of Nanog that is required for the transition from pre-iPSCs to iPSCs.

Induced pluripotency by defined factors is a very inefficient process. A series of studies indicate that critical tumor suppressors such as p53 and ARF are major checkpoints in suppressing induced pluripotency (Figure 3) [108]. The critical tumor suppression activity of p53 is underscored by the finding that p53 is inactivated in most human cancers either by gene mutation or the disruption of pathways required for p53 activation [109]. p53 is a transcription factor that directly regulates the expression of hundred of genes. For example, p53 directly activates the expression of genes involved in cell cycle arrest (p21, 14-3-3σ), apoptosis (Puma, Noxa) and senescence (PAI-1), and suppresses the expression of genes such as MAP4 and Nanog [110]. In addition to p53-dependent transcription, p53 also plays important transcription-independent roles in physiological processes such as metabolism [111] and miRNA processing [112].

In the absence of any stresses, p53 is inactive and unstable. Once activated in response to genotoxic and oncogenic stresses, p53 induces cell cycle arrest, apoptosis, or senescence in somatic cells [113, 114]. In ESCs, p53 plays a unique role in maintaining their genome stability. Upon DNA damage, p53 suppresses the expression of the pluripotency factor Nanog and thus induces the differentiation of ESCs harboring DNA damage [115]. This mechanism ensures that the self-renewing ESCs harbor no DNA damage, and thus are genetically stable. In support of an important role of p53 in maintaining genomic stability in ESCs, p53-deficient human ESCs exhibit extensive genomic instability [116]. The role of p53 in suppressing Nanog expression could also account for the findings that the silencing of p53 at the late stages of reprogramming of iPSCs increases the reprogramming efficiency because Nanog is important to promote the transition from pre-iPSCs to stable iPSCs [117]. It is also consistent with earlier findings that p53 is activated during ESC differentiation to inhibit the dedifferentiation by suppressing the Nanog expression [118].

In addition to c-Myc and Klf4 that are well-established oncogenes, other reprogramming factors including Oct4, Sox2, Nanog and Lin28 appear to have oncogenic potential [77]. In this context, Oct4, Sox2 and Nanog are frequently overexpressed in many types of human cancers and are correlated with the poor prognosis of the cancer patients [119–123]. The oncogenic stresses induced by the reprogramming factors can activate p53 [114]. In support of this notion, ARF, which is responsible for activating p53 in response to oncogenic stresses, also suppresses induced pluripotency [124–126]. In addition, the DNA double-stranded break damage induced during reprogramming, a potential outcome of oxidative stresses, can also activate p53 [124]. In support of this notion, DNA damage-induced activation of p53 is important to suppress induced pluripotency [127]. The activation of p53 leads to cell cycle arrest, apoptosis and senescence, any of which can block successful reprogramming. Therefore, p53 might be inactivated at least temporarily for the successful iPSC reprogramming. MdmX functions as an E3 ligase of p53, thus negatively regulates p53 activity. Stabilization of MdmX by mutation of three serine residues to alanines (Mdmx Ser 341, Ser 367 and Ser 402) dramatically decreases p53 activity and increases reprogramming efficiency [126] (Figure 3). Consistent with this notion, transient silencing of p53 can significantly increase the reprogramming efficiency [83, 124, 128] and recent studies have shown that Vitamin C can increase the reprogramming efficiency partly by inhibiting the ARF/p53 activation during reprogramming [129]. While the impact of the transient silencing of p53 on the genomic stability of iPSCs remains to be examined, the iPSCs derived from p53 null cells exhibit extensive genomic instability [128].

p53 plays multiple roles in tumor suppression. Therefore, it is important to understand which p53-dependent function is involved in suppressing induced pluripotency. Silencing of p21, which is required for p53-dependent cell cycle arrest [130], increases the reprogramming efficiency, indicating that p53-dependent cell cycle arrest is involved in suppressing induced pluripotency. The involvement of p53-dependent apoptosis in suppressing induced pluripotency is more complex. Puma, which is required for p53-dependent apoptosis after genotoxic stresses, is involved in suppressing induced pluripotency only when c-Myc is left out of the reprogramming cocktail [127]. This could be due to the findings that c-Myc significantly reduces the levels of oxidative stresses during the reprogramming [93], and high levels of oxidative stresses induce p53-dependent apoptosis [131]. Interestingly, in contrast to the greatly increased reprogramming-induced DNA damage in p53-deficient cells, the reprogramming-induced DNA damage in Puma-/-p21-/- cells is the same as the wild-type cells due to the increased senescence [127]. This raises the possibility that the transient silencing of Puma and p21 can increase the reprogramming efficiency of iPSCs without promoting genetic instability.

## Future perspective

While significant progress has been achieved to improve the reprogramming efficiency of iPSCs and reduce their cancer risk with new approaches to generate integration-free iPSCs, recent discoveries of the epigenetic and genetic abnormalities in iPSCs and the surprising immunogenicity of iPSC derivatives have raised safety concerns for clinic development of iPSCs. Considering the critical roles of p53 in maintaining genomic stability, it is important to elucidate which p53-dependent functions are involved in suppressing induced pluripotency. The acquired information can help to develop new strategy to retain the tumor suppression activity of p53 during the reprogramming into induced pluripotency. In addition, the reprogramming approach needs to be optimized to eliminate the components from the reprogramming cocktail that are involved in p53 inactivation. It is also important to resolve the bottleneck associated with the epigenetic abnormalities of iPSCs. Based on the findings that the epigenetics of the pluripotent stem cells generated by SCNT are more similar to ESCs [98], it is possible to optimize the reprogramming strategy to minimize the epigenetic difference between iPSCs and ESCs. In this context, small molecule compounds that can promote reprogramming efficiency by targeting epigenetic enzymes could help to achieve this goal. The overcome of these bottlenecks could also reduce the immunogenicity of iPSC-derived cells and improve the feasibility to develop iPSC-based human therapy.
